# Stereotactic body radiotherapy of central lung tumours using a 1.5 T MR-linac: First clinical experiences

**DOI:** 10.1016/j.ctro.2024.100744

**Published:** 2024-02-15

**Authors:** L.G. Merckel, J. Pomp, S.L. Hackett, A.L.H.M.W. van Lier, M. van den Dobbelsteen, M.J.A. Rasing, F.A.A. Mohamed Hoesein, L.M.W. Snoeren, C.A. van Es, P.S.N. van Rossum, M.F. Fast, J.J.C. Verhoeff

**Affiliations:** a Department of Radiotherapy, University Medical Center Utrecht, Heidelberglaan 100, 3584 CX Utrecht, The Netherlands; b Department of Radiology, University Medical Center Utrecht, Utrecht, The Netherlands

**Keywords:** MR-Linac, SBRT, (Ultra) central lung tumour

## Abstract

**Background:**

MRI-guidance may aid better discrimination between Organs at Risk (OARs) and target volumes in proximity of the mediastinum. We report the first clinical experiences with Stereotactic Body Radiotherapy (SBRT) of (ultra)central lung tumours on a 1.5 T MR-linac.

**Materials and Methods:**

Patients with an (ultra)central lung tumour were selected for MR-linac based SBRT treatment. A T2-weighted 3D sequence MRI acquired during free breathing was used for daily plan adaption. Prior to each fraction, contours of Internal Target Volume (ITV) and OARs were deformably propagated and amended by a radiation oncologist. Inter-fractional changes in volumes and coverage of target volumes as well as doses in OARs were evaluated in offline and online treatment plans.

**Results:**

Ten patients were treated and completed 60 Gy in 8 or 12 fractions. In total 104 fractions were delivered. The median time in the treatment room was 41 min with a median beam-on time of 8.9 min. No grade ≥3 acute toxicity was observed. In two patients, the ITV significantly decreased during treatment (58 % and 37 %, respectively) due to tumour shrinkage. In the other patients, 81 % of online ITVs were within ±15 % of the volume of fraction 1. Comparison with the pre-treatment plan showed that ITV coverage of the online plan was similar in 52 % and improved in 34 % of cases. Adaptation to meet OAR constraints, led to decreased ITV coverage in 14 %.

**Conclusions:**

We describe the workflow for MR-guided Radiotherapy and the feasibility of using 1.5 T MR-linac for SBRT of (ultra) central lung tumours.

## Introduction

Stereotactic body radiotherapy (SBRT)^2^ plays an important role in the radical treatment of small lesions like early-stage non-small cell lung cancer (NSCLC) or oligometastases from solid tumours [1], [2]. The introduction of 4D-Computed Tomography (CT) scans made it possible to irradiate moving lesions in the thorax and upper abdomen [3]. SBRT of lesions in the lung is generally well tolerated with limited toxicity and good local control, especially if a biological effective dose (BED) of >100 Gy can be applied [4], [5], [6]. Treatment of central or ultra-central lesions with high radical doses can result in severe toxicity [4]. Accepting reduced planning target volume (PTV) coverage, or a lower dose per fraction to avoid exceeding dose constraints of OARs, however, can impair the tumour control probability [7].

Magnetic resonance imaging (MRI) offers excellent soft-tissue contrast and has the potential to improve the delineation accuracy of mediastinal organs-at-risk (OARs) and target volumes compared to CT [8]. For tumours located close to the mediastinum, MRI can facilitate delineation by better distinction between tumour and OARs, and helps reducing the inter-observer contouring variability [9], [10]. Combining an MRI scanner with a linear accelerator (Linac) improves online visualisation of the target and OARs during treatment [11]. The MR images acquired before each treatment session visualise the patients’ daily anatomy and allows to create a treatment plan adapted to inter-fractional changes [12].

To date, few early experiences have been published on stereotactic MRI-guided radiotherapy for lung tumours. All reported treatments were performed with the MRIdian-system (Viewray Inc., Cleveland, USA), combining a Cobalt-60 or 6 MV Linac system with a 0.35 Tesla (T) MRI scanner [13]. In 2018, the 1.5 T MR-linac (Unity, Elekta AB, Stockholm, Sweden) became clinically available, combining a ring-based gantry containing a 7 MV Linac with a 1.5 T MRI scanner [11]. This system is increasingly used in clinical practice, mainly for treatments in the pelvic or abdominal region [14]. To the best of our knowledge, this is the first study reporting on the feasibility of SBRT of (ultra)centrally located tumours of the lung, in contact with the mediastinum on the 1.5 T MR-linac.

## Materials and Methods

### Patients

Patients with primary (non)-small cell lung cancer (NSCLC) and patients with oligo-progressive disease metastatic to the lung or mediastinum were eligible for treatment on the MR-Linac. Herder criteria were applied to establish malignancy with >90 % accuracy [15] MRI-guided treatment was offered if their planning target volume (PTV) was overlapping a 2 cm isotropic expansion of any critical mediastinal structure, including the bronchial tree, oesophagus, heart or major vessels. All patients provided written informed consent for use of their data as part of an ethics review board approved observational study (NCT04075305).

### Pre-treatment imaging, planning and quality assurance

Pre-treatment imaging included a CT scan (Brilliance CT big bore, Philips Medical Systems, Best, the Netherlands) and MRI scan on a 1.5 T Philips Ingenia MRI scanner for simulation. Respiratory triggered scans were acquired but not used for treatment planning. The free breathing 3D MRI scans acquired during each treatment (IntraMRI), as well as respiration-triggered scans, comprised a T2-weighted contrast.[16]. Both CT and MRI scans were performed in treatment position with the arms down to ensure patient comfort. A vacuum mattress (Blue-BAG, Elekta AB, Stockholm, Sweden) was used for comfort if necessary. The MRI scans were rigidly registered to CT and – if available – ^18^F-FDG positron emission tomography (PET)/CT scans. A dedicated lung radiation oncologist delineated the gross tumour volume (GTV), where after an Internal Target Volume (ITV) was constructed using the 4D-CT to take into account the breathing motion. It was verified that the free-breathing simulation MRI scan correctly visualized the ITV. An isotropic margin of 3 mm was added to the ITV to create the PTV akin to current institutional clinical practice when using CBCT-linac. Relevant mediastinal OARs (heart, oesophagus, trachea, main stem bronchi, and aorta) as well as lungs and spinal cord were contoured. The brachial plexus was contoured for dose calculation on the planning CT if located within 2 cm from the ITV [17], [18]. A 3D sphere of 3 cm around the ITV was generated inside which delineations were adapted online by the radiation oncologist.

An average CT was generated by averaging over all breathing phases, and was used for treatment planning. To assign a relative electron density (RED) to the PTV, the average density in the GTV was forced on the full PTV [19]. The bulk electron densities of each main bronchus, each lung, the trachea and bones were based on the average densities of these structures on the pre-treatment CT scan. All other tissues were assigned a relative electron density of 1.0. For each patient, a pre-treatment step-and-shoot intensity-modulated radiotherapy (IMRT) plan was created in Monaco v5.40.01 (Elekta AB, Stockholm, Sweden), which included simulation of beam characteristics of the linear accelerator, the magnetic field, the posterior coil and the treatment couch of the MR-linac [11]. Ten to 15 beam angles were used, and beam angles were selected to avoid the high-density couch rails and the cryostat pipe, and to limit beam entry through the patient’s arms [20]. A grid size of 3 mm was used for dose calculations, and the maximum number of segments was set to 45. Four patients received 8 fractions of 7.5 Gy (BED_10_ = 105) and 6 patients received 12 fractions of 5 Gy (BED_10_ = 90). The aim was a coverage of 100 % of the ITV (V_60Gy_ = 100 %) and a coverage of at least 95 % of the PTV (V_60Gy_ ≥95 %). Planning was performed using a steep, stereotactic dose gradient with a maximum dose of 145 % of the prescription dose allowed within the GTV. Target coverage was evaluated on the ITV, [21]. Quality assurance measurements of all pre-treatment plans were performed with an MR-compatible Delta4 phantom, or with GafChromic EBT3 film (Ashland Inc. Wayne, NJ, USA.) if the PTV was less than 5 cc. The pre-treatment plan was used as a patient-specific template for online treatment planning.

### Plan evaluation

Coverage of ITV and PTV, as well as the dose in OARs were evaluated, and compared with the delineation and dose calculation on the planning CT scan. The OAR constraints that were used are added in the Supplementary Materials (Table). The choice to prioritize PTV coverage or fulfilment of OAR constraints was at the discretion of the treating physician. ICRU guideline for SBRT was used for ITV coverage [21]. The daily online treatment plan was based on the T2-weighted MRI used for online delineation and planning (PreMRI). The post-treatment plan was this same plan recalculated on the T2-weighted MRI scanned just after the beam was off (PostMRI). When comparing the initial pre-treatment plan as calculated on the pre-treatment 4D CT scan, with the daily online adapted treatment plan regarding ITV and PTV coverage, a difference of >1 % was considered to be clinically relevant. Maximum doses to 0.1 cm^3^ (D0.1 cc) of OARs were collected and converted to Equivalent dose of 2 Gy fractions (EQD_2_) by applying an α/β of 3 Gy for the OARs and 2 Gy for the spinal cord [22], [23].

### MRI-guided online workflow for lung tumours

Patients were positioned on the MRL couch using specific couch index points and lasers [24]. An in-house developed T2-weighted 3D MRI sequence acquired during free breathing, was used for daily online delineation and treatment planning (TR 1000 ms, TE 100 ms, acquired voxel size 1.2 × 1.2 × 2.4 mm^3^, reconstructed voxel size 0.59 × 0.59 × 2.4 mm^3^, FOV 350 [AP] × 451 [LR] × 180 [CC] mm^3^). Each treatment session started with the acquisition of an MRI scan, the PreMRI, followed by a Position Verification MRI (PVMRI) which is acquired while the contours on the PreMRI are modified and treatment dose is recalculated. The PVMRI was then imported into the planning system for position verification. During the irradiation the third MRI was acquired, the IntraMRI, and after the irradiation the fourth and final MRI was made; the PostMRI. Post-treatment imaging was performed using the same T2-weighted 3D sequence used for online delineation and position verification. For the first fraction in each patient, contours were automatically propagated from the pre-treatment CT to the PreMRI using deformable image registration. For all other fractions, contours from the PVMRI scan of the first fraction were propagated to the next PreMRI. The contour propagation was visually checked, and we took advantage of the contrast being exactly the same for the position verification MRI of the first fractions and the PreMRI of the sequential sessions, allowing for improved and consistent contour propagation For all fractions, the adapt to shape (ATS) workflow was used in which online plan adaptation is performed on the patients’ anatomy at time of treatment and optimized on the adapted contours on the PreMRI [25]. The treating physician could choose an additional adapt to position (ATP) procedure if deemed necessary when a shift of more than 1.5 mm of the ITV was seen between PreMRI and PVMRI, whereby the segments of the treatment plan are shifted to the new position of the ITV on the PVMRI. For the ATP procedure, online plan adaptation was performed by rigidly registering the PreMRI with the PVMRI and optimizing the plan to account for the change of patient position. An additional ATS procedure could be performed as well when deemed necessary due to changes in posture. The treatment plan was delivered in free-breathing after approval by the treating physician. Quality assurance measurements were performed for the first online plan for each patient, using the same phantom as used to measure the pre-treatment plan for the patient.

### Follow-up

Patients were seen 4 weeks after completion of the radiotherapy by their treating physician where after they returned to their pulmonologist or medical oncologist for subsequent follow-up. The follow-up generally consisted of routine CT imaging every 3 months in the first year.

## Results

Between December 2019 and October 2020, 10 patients were treated with SBRT for a tumour in the lung or mediastinum using the MRL. Median age of patients was 59 years (range 45–79) and 60 % of patients were female. Two patients had NSCLC, 1 patient had SCLC, and 2 patients had no histologically proven lesion but their tumours were considered to be early-stage NSCLC. Five patients were treated for oligometastases from different origins (oropharynx, colon, breast, kidney and larynx). One patient with a squamous cell carcinoma of the right lung was treated with SBRT for an infield recurrence 2 years after 3 Gy × 13. Four patients had a central tumour and were treated with 7.5 Gy × 8, six patients had an ultra-central tumour located in or abutting the mediastinum and were treated with 5 Gy × 12. Two patients were treated on 2 separate PTVs to treat local tumour and regional lymph nodes simultaneously. A median of 5 OARs per patient, (range 1–8), were located within 3 cm around the ITV (Table 1).

**Table 1:** Frequency of OARs requiring contour verification and adjustment online (i.e. within 3 cm from the ITV).

OAR	Frequency
Trachea	8/10
Spinal cord	7/10
Heart	5/10
Oesophagus	9/10
Main Bronchus	7/10
Aorta	8/10
Brachial Plexus	2/10

All but one patient (with 2 PTVs; tumour and lymph node separately) started their treatment within two weeks after the pre-treatment planning CT scan. The interval for the patient with 2 PTVs was 26 days. All ten patients completed their planned fractions on the MRL using the ATS workflow. For the one patient with two separate PTVs, a second ATS procedure was necessary three out of 12 times, due to change in relative position and spacing between the two PTVs. On these occasions, a deformable registration between the PreMRI and the PVMRI was performed, and the plan was adapted to the anatomy accordingly. An additional ATP was performed 6 times in 3 different patients because the PVMRI showed a displacement of >1.5 mm. All pre-treatment plans showed a gamma pass rate of ≥99.3 %, calculated using a 3 %/3 mm criterion. For the measured online plans, all gamma pass rates were ≥98.8 %.

**Fig. 1:**
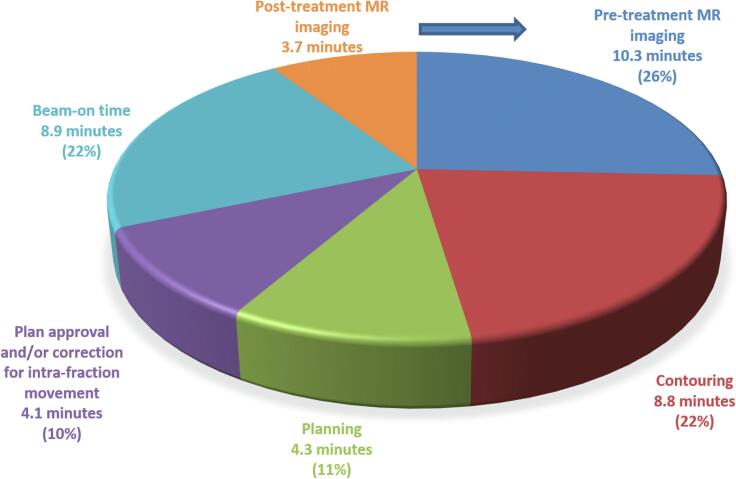
Duration of MRI-guided lung SBRT workflow clockwise starting with pre-treatment MR imaging. The PVMRI and IntraMRI were acquired in parallel with other workflow steps and did not require additional time.

The median duration of the total treatment procedure, as measured from the start of PreMRI to the end of the PostMRI, was 41 min (5–95th percentile: 34–58 min). The median beam-on time was 8.9 min (5–95th percentile: 7.3–12.3 min), accounting for 22 % of the duration of the total procedure (Fig. 1). The median number of beams and segments was 13 (range 10–15) and 44 (range 41–45).

The planning ITV ranged from 1.5 to 148 cm^3^ (median 14.9 cm^3^). Five ITVs were smaller than 10 cm^3^, 4 ITVs were between 10 and 30 cm^3^ and 3 ITVs over 100 cm^3^. Median planning PTV size was 27.4 cm^3^ (range 4.6–201). In 75 % of the ITVs, the volume on the PreMRI of the first fraction was on average 6.5 % larger compared to the pre-treatment ITV. Both the ITVs in the patient with 26 days interval, had grown more than 25 % since the pre-treatment scans.

**Fig. 2:**
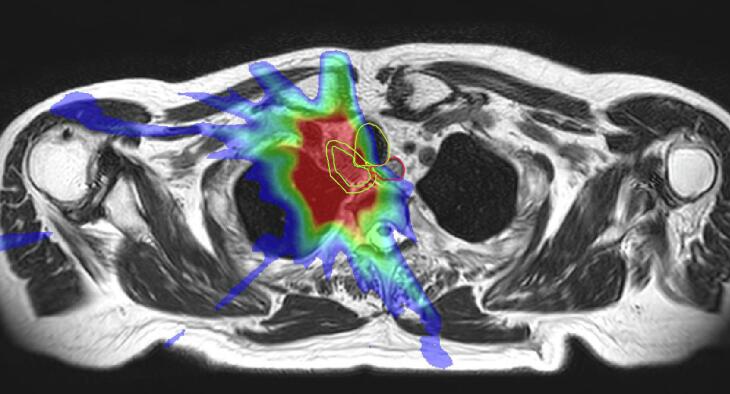
Online MRI and physical dose distribution of a patient with a mediastinal lymph node metastasis of a SCLC in whom a 58 % decrease in ITV occurred. The dose distribution of the original plan is projected on the reduced target size at the 12th fraction The ITV (inner green line) and OARs (oesophagus, trachea, spinal cord) within a 3 cm expansion (yellow) around the ITV were modified each fraction by a radiation oncologist.

In two patients, a significant decrease in ITV during treatment of 58 % (primary SCLC) (Fig. 2), and 37 % occurred due to tumour shrinkage. In the other patients, 81 % of online ITVs remained within ±15 % of the volume of fraction 1. Median online planning coverage was 99.3 % (range 80.3–100) for ITV and 89.7 % (65.6–98.1) for PTV. For 5 ITVs in 4 patients, the ITV coverage during all fractions was 99.5 % or higher. For 7 ITVs in the other 6 patients, a lower coverage of ITV and PTV was accepted because the treating physician prioritized the dose in OARs over the ITV and PTV coverage. Median online ITV and PTV coverage on the daily online plan were 95.8 % (range 73.1–100) and 87.2 % (range 55.8–99.7). When comparing to the pre-treatment planning CT plan, ITV coverage of the daily online treatment plan was similar in 52.3 %, better in 33.6 % and decreased in 14.1 % of fractions. PTV coverage was similar in 23.4 %, better in 44.5 % and decreased in 32.0 % of fractions. Median online ITV and PTV coverage on the post-treatment online plan were 94.1 % (range 61.7–100) and 82.3 % (range 49.7–98.8). Maximum doses in the OARs were comparable between the pre-treatment and daily online plans. In 2 patients the maximum cardiac dose and dose to 15 cc of the heart was exceeded, and accepted for optimal PTV coverage. The brachial plexus was always more than 1 cm away from the PTV and dose constraints were never exceeded. No grade ≥3 acute toxicity was observed during treatment and the first four weeks after treatment. With a follow up time of 30 months, 5 of 10 patients have died, 7–22 months after treatment. The other 5 patients are still alive 30–36 months after treatment. Two patients (20 %), both treated with 12 fractions of 5 Gy, developed an in-field recurrence; one with reirradiation indication for locally recurrent squamous cell carcinoma of the right lung treated 18 months earlier with 3 Gy × 13, and one patient, treated for a metastasis from oropharynx carcinoma developed local recurrence after 6 months.

## Discussion and future

This feasibility study involved a heterogeneous group of 10 patients with primary (5) or secondary (5) lung tumours, with 1 or 2 ITVs and including 1 re-irradiation. The BED_10_ that was applied was 90 Gy (6 times), or 105 Gy (4 times). The small number and the heterogeneity of indications applied, does not allow to draw conclusions with regard to clinical indications and guidelines. To date, no randomized trials exist comparing the treatment outcomes, after SBRT on the MR-Linac with the outcomes of SBRT treatments on a cone-beam CT-linac (cbct)-linac. For tumours located near, or attached to critical organs, MRI images provide an excellent soft tissue contrast and are superior to the image quality of the CBCT. For treatment with CBCT-Linac, the uncertainty to discriminate a centrally located tumour from its surrounding OARs, leads to reduced dose per fraction while applying increased margins, resulting in worse BED_10_. The longer treatment time on the MRLinac can be negated by the reduced number of sessions compared to treatment on a CBCT-Linac. Improved visualisation during treatment will most likely lead to more accurate targeting of the tumour with better sparing of OAR allowing for higher dose per fraction. Accrual for a randomized trial comparing two different imaging techniques is doomed to fail for tumours that are better recognized on an MRI than on a CBCT. Close follow-up and consciously scoring of toxicity, local control and OS, will enable the comparison with historic data from treatment on CBCT-linacs. Yan et al. recently published a review about the outcome after SBRT for ultracentrally located tumours and estimated from literature an average 6 % grade 3–4 toxicity while treatment related death occurred in 4 %. Recently published data of single fraction MR guided SBRT in 50 patients showed toxicity comparing favourably with historical SBRT data; only 1/50 patients developed grade ≥3 toxicity and local control was 97 % [21], [26].

Multiple developments will further improve treatments of moving tumours on the MR-linac with regard to speed, efficiency and reliability. Today, a CT scan is part of the workflow for delineation and planning. With Deep Learning (DL) generated artificial CT, it will become possible to omit the CT scan, and use only MRI scans for delineation, planning, and treatment [27]. MR-linacs support several intra-fractional respiratory motion mitigation techniques, enabling SBRT in lung and the upper abdomen. With daily respiratory-correlated 4D-MR imaging respiratory-induced motion of the tumour and OARS can be estimated and mitigated using either an ITV or mid-position planning approach [14], [28], [29]. After the inclusion period of this study, comprehensive motion management became available on the 1.5 T MR-linac which includes respiratory gating options and baseline motion corrections through trailing [30], [31]. In the future, multi-leaf collimator (MLC) tracking which combines the dosimetric precision of gating with a 100 % delivery duty cycle, might become more readily available on the MR-linac [32].

## Conclusions

We demonstrate the feasibility of SBRT of (ultra)centrally located lung tumours on a 1.5 T MRL and show that daily MR-guided plan adaptation is advantageous in terms of target coverage or OAR sparing. Tumours located close to critical structures, or recurrent tumours after previous radiotherapy when tolerance dose for OAR have been reached may profit from treatment on an MR-Linac. Ongoing technical developments are expected to further increase these benefits.

## CRediT authorship contribution statement

**L.G. Merckel:** Writing – original draft, Visualization. **J. Pomp:** Writing – original draft. **S.L. Hackett:** Conceptualization, Methodology, Writing – review & editing. **A. van Lier:** Methodology, Conceptualization. **M. van den Dobbelsteen:** Methodology. **M.J.A. Rasing:** Methodology. **F.A.A. Mohamed Hoesein:** Writing – review & editing. **L.M.W. Snoeren:** Supervision. **C.A. van Es:** Review and editing. **P.S.N. van Rossum:** Writing – review & editing. **M.F. Fast:** Methodology, Writing – review & editing, Supervision, Project administration. **J.J.C. Verhoeff:** Conceptualization, Writing – review & editing, Supervision, Project administration.

## Declaration of competing interest

The authors declare that they have no known competing financial interests or personal relationships that could have appeared to influence the work reported in this paper.

## Footnotes

2 Abbreviations; PreMRI, Initial MRI at start treatment; PVMRI, Position verification MRI; IntraMRI, MRI generated during irradiation (beam on mode); PostMRI, post-treatment MRI imaging after beam stopped; DL, Deep Learning

## References

[1] Hiraoka M., Matsuo Y., Nagata Y. (2007). Stereotactic body radiation therapy (SBRT) for early-stage lung cancer. Cancer/radiotherapie.

[2] Mayinger M., Kotecha R., Sahgal A., Kim M.-S., Lo S.S., Louie A.V. (2023). Stereotactic body radiotherapy for lung oligo-metastases: systematic review and international stereotactic radiosurgery society practice guidelines. Lung Cancer.

[3] Dhont J., Harden S.V., Chee L.Y.S., Aitken K., Hanna G.G., Bertholet J. (2020). Image-guided radiotherapy to manage respiratory motion: lung and liver. Clin Oncol.

[4] Yan M., Louie A.V., Kotecha R., Ashfaq Ahmed M., Zhang Z., Guckenberger M. (2023). Stereotactic body radiotherapy for Ultra-Central lung Tumors: A systematic review and Meta-Analysis and International Stereotactic Radiosurgery Society practice guidelines. Lung Cancer.

[5] Li F., Jiang H., Bu M., Mu X., Zhao H. (2022). Dose-effect relationship of stereotactic body radiotherapy in non-small cell lung cancer patients. Radiat Oncol.

[6] Kutuk T., Herrera R., Mustafayev T.Z., Gungor G., Ugurluer G., Atalar B. (2022). Multi-institutional outcomes of stereotactic magnetic resonance image guided adaptive radiation therapy with a median biologically effective dose of 100 Gy10 for non-bone oligometastases. Adv Radiat Oncol.

[7] Zhao Y., Khawandanh E., Thomas S., Zhang S., Dunne E.M., Liu M. (2020). Outcomes of stereotactic body radiotherapy 60 Gy in 8 fractions when prioritizing organs at risk for central and ultracentral lung tumors. Radiat Oncol.

[8] Keall P.J., Brighi C., Glide-Hurst C., Liney G., Liu P.Z.Y., Lydiard S. (2022). Integrated MRI-guided radiotherapy — opportunities and challenges. Nat Rev Clin Oncol.

[9] Bainbridge H., Salem A., Tijssen R.H.N.N., Dubec M., Wetscherek A., Van Es C.V. (2017). Magnetic resonance imaging in precision radiation therapy for lung cancer. Transl Lung Cancer Res.

[10] Goodburn R.J., Philippens M.E.P., Lefebvre T.L., Khalifa A., Bruijnen T., Freedman J.N. (2022). The future of MRI in radiation therapy: Challenges and opportunities for the MR community. Magn Reson Med.

[11] Raaymakers B.W., Jürgenliemk-Schulz I.M., Bol G.H., Glitzner M., Kotte A.N.T.J., Van Asselen B. (2017). First patients treated with a 1.5 T MRI-Linac: Clinical proof of concept of a high-precision, high-field MRI guided radiotherapy treatment. Phys Med Biol.

[12] Chetty I.J., Doemer A.J., Dolan J.L., Kim J.P., Cunningham J.M., Dragovic J. (2022). MRI-guided Radiotherapy (MRgRT) for treatment of oligometastases: review of clinical applications and challenges. Int J Radiat Oncol Biol Phys.

[13] Klüter S. (2019). Technical design and concept of a 0.35 T MR-Linac. Clin Transl Radiat Oncol.

[14] Paulson E.S., Ahunbay E., Chen X., Mickevicius N.J., Chen G.P., Schultz C. (2020). 4D-MRI driven MR-guided online adaptive radiotherapy for abdominal stereotactic body radiation therapy on a high field MR-Linac: Implementation and initial clinical experience. Clin Transl Radiat Oncol.

[15] Herder G.J., Van Tinteren H., Golding R.P., Kostense P.J., Comans E.F., Smit E.F. (2005). Clinical prediction model to characterize pulmonary nodules: Validation and added value of18F-fluorodeoxyglucose positron emission tomography. Chest.

[16] Thorwarth D., Low D.A. (2021). Technical challenges of real-time adaptive MR-guided radiotherapy. Front Oncol.

[17] De Rose F., Franceschini D., Reggiori G., Stravato A., Navarria P., Ascolese A.M. (2017). Organs at risk in lung SBRT. Phys Medica.

[18] Ing F.E.N.G., Ong S.P.K., Itter T.I.R., Uint D.O.J.Q., Enan S.U.S., Aspar L.A.E.G. (2011). Consideration of dose limits for organs at risk of thoracic radiotherapy: Atlas for lung, proximal bronchial tree, esophagus, spinal cord, ribs, and brachial plexus. Int J Radiat Oncol Biol Phys.

[19] Schrenk O., Spindeldreier C.K., Schmitt D., Roeder F., Bangert M., Burigo L.N. (2018). The effect of density overrides on magnetic resonance-guided radiation therapy planning for lung cancer. Phys Imaging Radiat Oncol.

[20] van den Wollenberg W., de Ruiter P., Nowee M.E., Jansen E.P.M., Sonke J.J., Fast M.F. (2019). Investigating the impact of patient arm position in an MR-linac on liver SBRT treatment plans. Med Phys.

[21] de Jong E.E.C., Guckenberger M., Andratschke N., Dieckmann K., Hoogeman M.S., Milder M. (2020). Variation in current prescription practice of stereotactic body radiotherapy for peripherally located early stage non-small cell lung cancer: Recommendations for prescribing and recording according to the ACROP guideline and ICRU report 91. Radiother Oncol.

[22] Scheenstra A.E.H., Rossi M.M.G., Belderbos J.S.A., Damen E.M.F., Lebesque J.V., Sonke J.J. (2014). Alpha/beta ratio for normal lung tissue as estimated from lung cancer patients treated with stereotactic body and conventionally fractionated radiation therapy. Int J Radiat Oncol Biol Phys.

[23] Schultheiss T.E. (2008). The radiation dose-response of the human spinal cord. Int J Radiat Oncol Biol Phys.

[24] Werensteijn-Honingh A.M., Kroon P.S., Winkel D., Aalbers E.M., van Asselen B., Bol G.H. (2019). Feasibility of stereotactic radiotherapy using a 1.5 T MR-linac: Multi-fraction treatment of pelvic lymph node oligometastases. Radiother Oncol.

[25] Winkel D., Bol G.H., Kroon P.S., van Asselen B., Hackett S.S., Werensteijn-Honingh A.M. (2019). Adaptive radiotherapy: The Elekta Unity MR-linac concept. Clin Transl Radiat Oncol.

[26] Tekatli H, Palacios MA, Schneiders FL, Haasbeek CJA, Slotman BJ, Lagerwaard FJ, et al. Local control and toxicity after magnetic resonance imaging (MR)-guided single fraction lung stereotactic ablative radiotherapy. Radiother Oncol 2023;187. https://doi.org/10.1016/j.radonc.2023.109823. 10.1016/j.radonc.2023.109823 37516364

[27] Jonsson J., Nyholm T., Söderkvist K. (2019). The rationale for MR-only treatment planning for external radiotherapy. Clin Transl Radiat Oncol.

[28] Keijnemans K., Borman P.T.S., Uijtewaal P., Woodhead P.L., Raaymakers B.W., Fast M.F. (2022). A hybrid 2D/4D-MRI methodology using simultaneous multislice imaging for radiotherapy guidance. Med Phys.

[29] van de Lindt T.N., Nowee M.E., Janssen T., Schneider C., Remeijer P., van Pelt V.W.J. (2022). Technical feasibility and clinical evaluation of 4D-MRI guided liver‘2 SBRT on the MR-linac. Radiother Oncol.

[30] Fast M., van de Schoot A., van de Lindt T., Carbaat C., van der Heide U., Sonke J.J. (2019). Tumor Trailing for Liver SBRT on the MR-Linac. Int J Radiat Oncol Biol Phys.

[31] Grimbergen G, Hackett SL, van Ommen F, van Lier ALHMW, Borman PTS, Meijers LTC, et al. Gating and intrafraction drift correction on a 1.5 T MR-Linac: Clinical dosimetric benefits for upper abdominal tumors. Radiother Oncol 2023;189:109932. https://doi.org/10.1016/j.radonc.2023.109932. 10.1016/j.radonc.2023.109932 37778533

[32] Uijtewaal P., Borman P.T.S., Woodhead P.L., Hackett S.L., Raaymakers B.W., Fast M.F. (2021). Dosimetric evaluation of MRI-guided multi-leaf collimator tracking and trailing for lung stereotactic body radiation therapy. Med Phys.

