# Supplementary material for: Stereotactic body radiotherapy of central lung tumours using a 1.5 T MR-linac: First clinical experiences
Source: Clin Transl Radiat Oncol. 2024 Feb 15;45:100744. doi: 10.1016/j.ctro.2024.100744 (PMC10885732; doi:10.1016/j.ctro.2024.100744)
Supplement: Supplementary Data 1 [file mmc1.docx]

# Supplementary data

|  |  |  |  |  |
| --- | --- | --- | --- | --- |
|  | **8 x 7,5 Gy**  **Hard** | **8 x 7,5 Gy**  **Soft** | **12 x 5 Gy**  **Hard** | **12 x 5 Gy**  **Soft** |
| **Organ at risk** |  |  |  |  |
| Aorta | V_49Gy_ < 0.5 cm^3^  V_30Gy_< 5 cm^3^ |  | V_58Gy_ < 0.5 cm^3^  V_36Gy_ < 5 cm^3^ |  |
| Spinal cord | V_33Gy_ <0.1 cm^3^ |  | V_38Gy_ <0.1 cm^3^ |  |
| Oesophagus | V_38Gy_ <0.5 cm^3^  V_31Gy_ <5 cm^3^ |  | V_44Gy_ <0.5 cm^3^  V_36Gy_ <5 cm^3^ |  |
| Trachea | V_42Gy_ <0.5 cm^3^ |  | V_49Gy_ <0.5 cm^3^ |  |
| Main left/right bronchus | V_42Gy_ < 0.5 cm^3^ |  | V_49Gy_ < 0.5 cm^3^ |  |
| Heart | V_49Gy_ < 0.5 cm^3^  V_37Gy_ Gy < 15 cm^3^ |  | V_58Gy_ < 0.5 cm^3^  V_42Gy_ < 15 cm^3^ |  |
| Lung minus ITV  D_mean_ Lung | V_20Gy_ Gy < 10%  < 20 Gy |  | V_20Gy_ < 10%  < 20 Gy |  |
| Skin |  | V_49Gy_ Gy < 0.5 cm^3^  V_44Gy_ Gy < 10 cm^3^ |  | V_58Gy_ < 0.5 cm^3^  V_57Gy_ < 10 cm^3^ |
| Brachial Plexus | V_38Gy_ Gy < 0.1 cm^3^ |  | V_43Gy_ Gy < 0.1 cm^3^ |  |

Supplementary Table 1s. Hard and Soft Constraints for OARs
